# Can sustained arousal explain the Chronic Fatigue Syndrome?

**DOI:** 10.1186/1744-9081-5-10

**Published:** 2009-02-23

**Authors:** Vegard B Wyller, Hege R Eriksen, Kirsti Malterud

**Affiliations:** 1 Division of Paediatrics, Rikshospitalet University Hospital, Oslo, Norway; 2 Department of Education and Health Promotion, University of Bergen, Bergen, Norway; 3 Unifob Health Bergen, Bergen, Norway; 4 Research Unit for General Practice, Unifob Health Bergen, Bergen, Norway

## Abstract

We present an integrative model of disease mechanisms in the Chronic Fatigue Syndrome (CFS), unifying empirical findings from different research traditions. Based upon the Cognitive activation theory of stress (CATS), we argue that new data on cardiovascular and thermoregulatory regulation indicate a state of permanent arousal responses – *sustained arousal *– in this condition. We suggest that sustained arousal can originate from different precipitating factors (infections, psychosocial challenges) interacting with predisposing factors (genetic traits, personality) and learned expectancies (classical and operant conditioning). Furthermore, sustained arousal may explain documented alterations by establishing vicious circles within immunology (Th2 (humoral) vs Th1 (cellular) predominance), endocrinology (attenuated HPA axis), skeletal muscle function (attenuated cortical activation, increased oxidative stress) and cognition (impaired memory and information processing). Finally, we propose a causal link between sustained arousal and the experience of fatigue. The model of sustained arousal embraces all main findings concerning CFS disease mechanisms within one theoretical framework.

## Background

*Chronic Fatigue Syndrome *(CFS) is characterized by unexplained and disabling fatigue, accompanied by symptoms such as musculoskeletal pain, impaired memory and concentration, headache and sleep problems [1]. Research on disease mechanisms has been conducted along several tracks (Table 1). Twin studies indicate a moderate heritability of CFS [2]. Recent molecular analyses report an association to polymorphisms of genes involved in autonomic and endocrine effector systems [3]. Personality traits such as perfectionism, conscientiousness and internalization may have an impact [4], as do illness perceptions such as a poor sense of personal control over symptoms and a strong focus on bodily sensations [5]. In many patients, firm evidence supports a relation to long-lasting infection caused by different microorganisms, such as Epstein-Barr virus, enteroviruses, and *Coxiella burnetii *[6,7]. In addition, CFS may be initiated by critical life events or perceived chronic difficulties [8,9].

**Table 1:** Summary of main findings related to disease mechanisms in Chronic Fatigue Syndrome

**Predisposing factors**
Genetic traits

Polymorphisms in autonomic and endocrine effector systems

Personality traits

Inappropriate illness perceptions

**Precipitating factors**

Long-lasting infections

Critical life events

Perceived chronic difficulties

**Perpetuating and associated factors**

Hemodynamic alterations

Sympathetic vs parasympathetic predominance

Immune alterations

Th2 vs Th1 predominance

Endocrine alterations

Attenuated HPA axis

Skeletal muscle alterations

Attenuated cortical activation

Increased oxidative stress

Cognitive alterations

Impaired memory and information processing

Regarding perpetuating and associated factors, hemodynamic disturbances characterized by increased sympathetic and attenuated parasympathetic cardiovascular neurotransmission have been documented [10,11]. Immune system alterations (Th2 vs Th1 immune response predominance) are also reported [2]. There is evidence for hypofunction of the HPA-axis in some CFS patients [12]. Reduced function of skeletal muscles [13] might be due to functional changes in cortical motor areas [14], but could also be caused by changes in muscle metabolism due to increased oxidative stress [15,16]. Finally, cognitive tests have revealed disturbances of memory and speed of information processing [17,18], but overall normal functioning in other cognitive domains.

A coherent and integrative model of CFS disease mechanisms combining these findings is lacking. In this article, we propose such a model, based upon the Cognitive activation theory of stress (CATS) [19]. Specifically, we suggest that CFS is caused by *sustained arousal*.

## The sustained arousal model of CFS

Below, we first present CATS – the stress theory upon which our model is based. Then we outline empirical indications of an association between sustained arousal and CFS. From this point of departure, we apply CATS to hypothesize the potential mechanisms leading to sustained arousal in CFS, and finally we substantiate our model by discussing sustained arousal consequences in other organ systems in relation to CFS research evidence.

### The Cognitive activation theory of stress (CATS)

Among various definitions of the term "stress", a common denominator is that stress denotes any condition being a threat to homeostasis in a broad sense [20,21]. Stress occurs whenever there is a discrepancy between what is expected ("set value") and what really exists ("actual value"); hence, it always implies comparison of present sensory information with stored brain information [19,22]. This may be a fast and partly automatic process, for instance when exposed to a significant and unexpected change in the environment [23], or when certain physiologic variables (such as blood pressure) are perturbated [20]. Eventually, the comparison may involve complex cognitive evaluations of situations and their potential consequences, which in turn is based on previous experiences in equal or similar situations [19,24].

**Figure 1:**
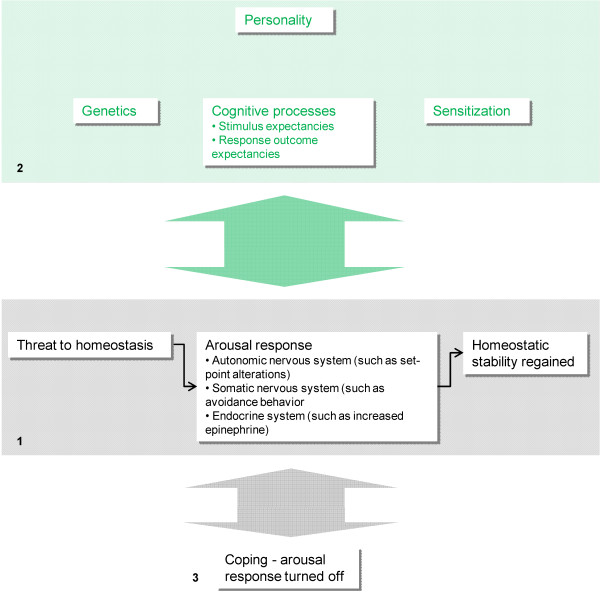
**Normal arousal response, according to the Cognitive activation theory of stress (CATS)**. A threat to homeostasis elicits an arousal response, characterized by nervous and endocrine adjustments aiming at regaining homeostatic stability (1). This compensatory mechanism is mutually connected to cognitive processes; in addition, it is influenced by personality, genetic traits and sensitization (2). If successful, i.e. if homeostasis is restored, the arousal response is turned off (3).

Stress normally elicits a quite non-specific *arousal response*, involving the somatic and autonomic nervous system as well as several endocrine axes [25] (Figure 1). Important characteristics include elevated plasma levels of epinephrine and a change of set-point in homeostatic control circuits (causing, for instance, elevated blood pressure and body temperature) [20]. The overall purpose of the arousal response is to restore homeostasis by counteracting the initial discrepancy between expectations and reality. The arousal response is gradually turned off when successful ("coping"). If not, the arousal may be sustained [19].

Although quite uniform in gross, the details and dynamics of the arousal response vary among individuals. Genetic variability has some impact [20], but cognitive processes, evaluating the relationships between stimulus and expectancy, may be more important to explain individual variation [22]. These mechanisms have been systematized in the *Cognitive activation theory of stress *(CATS) [19]. The theoretical basis of CATS is the cognitive reformulations of learning theory [24,26], where classical conditioning is regarded as acquisition of expectancies of the outcomes of stimuli (*stimulus expectancies*), and instrumental or operant conditioning as the acquisition of expectancies of the results of available responses (*response outcome expectancies*). Arousal response intensity increases if the stimulus expectancy has high affective value or if the response outcome expectancy is inadequate. Furthermore, experimental studies have demonstrated that arousal response can be modified by *sensitization*, the enhanced response to repeated stimulation [27]. This phenomenon has been described in detail on the cellular level [28], and is also considered important for disease development [29,30]. Thus, sensitization has been suggested as an important underlying mechanism in fibromyalgia [31], irritable bowel and functional dyspepsia [32], chemical intolerance, and somatization [30].

The arousal response is primarily adaptive and health-promoting. However, alterations of the response dynamics – in particular a state of maintenance, in CATS denoted *sustained arousal *– may contribute to disease [19,21].

### Indications of sustained arousal in CFS patients

The first author investigated cardiovascular and thermoregulatory homeostasis in adolescent CFS patients and healthy controls. During supine rest, CFS patients had increased sympathetic nerve activity to the heart, the skeletal muscle arterioles and the adrenals; the latter causing increased plasma levels of epinephrine. There was also evidence of increased arterial blood pressure and body temperature [33-35]. During orthostatic challenge, CFS patients demonstrated enhanced sympathetic nerve activity to the heart and the skeletal muscle arterioles, as well as increased arterial blood pressure [33-37]. However, when orthostatic challenge was combined with isometric exercise, CFS patients presented attenuated sympathetic cardiovascular outflow and a smaller increase in the arterial blood pressure [34]. During local cooling, CFS patients had attenuated sympathetic outflow to skin arterioles combined with normalization of body temperature [35]. Finally, CFS patients presented symptoms suggesting enhanced sympathetic nerve activity to the sweat glands, the skeletal muscles and the skin vessels [34,35].

Similar findings have previously been reported in other studies [10,11], and hypovolemia and deconditioning have been suggested as possible underlying mechanism. Yet, neither of these mechanisms can fully explain the results: Moderate hypovolemia usually does not cause altered blood pressure and body temperature [38], and deconditioning leads to attenuated sympathetic cardiovascular responses during orthostatic challenge [39]. Rather, the results suggest alterations of CNS autonomic control, corresponding with neuroimaging studies indicating functional alterations in relevant brain stem areas [40]. More specifically, the response patterns might all be explained by abnormalities in homeostatic set-point adjustments of blood pressure and body temperature [41]. For instance, abnormal increase in arterial blood pressure set-point during orthostatic challenge might enhance sympathetic nerve activity to the heart and the skeletal muscle arterioles, causing increased heart rate and total peripheral resistance and bringing the observed blood pressure value to a higher level.

According to stress theory, set-point changes of homeostatic control circuits are hallmarks of the arousal response, as is increased level of epinephrine [20], demonstrated in CFS patients by Wyller [35] and others [42]. Furthermore, Wyller and co-workers' results comply neatly with human and animal studies directly addressing cardiovascular and thermoregulatory alterations during arousal [43,44]. Thus, CFS patients seem to present an arousal response-physiology which is, however, inappropriate, being present both at rest and during maneuvers which are normally not distressing. We propose these findings to be interpreted as indications of sustained arousal in CFS patients.

### Potential origin of sustained arousal in CFS

**Figure 2:**
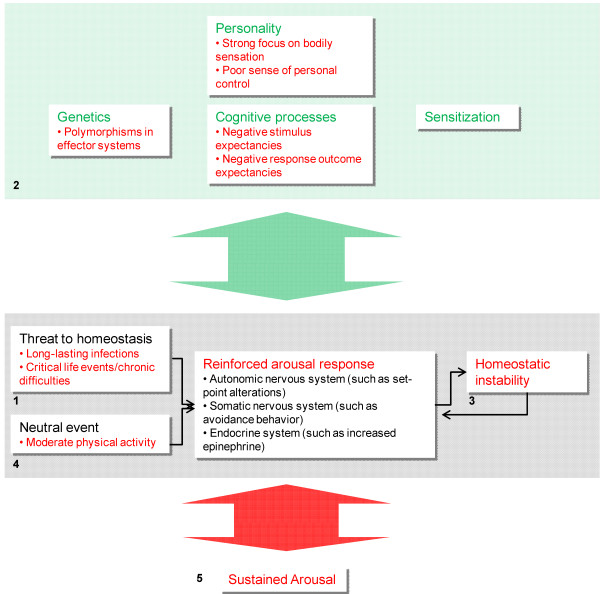
**Proposed model of the origin of sustained arousal in Chronic fatigue syndrome**. Certain threats to homeostasis, such as long-lasting infections and psychosocial challenges, may elicit a prolonged arousal response, which does not, however, solve the initial problem (1). The mutual relation to cognitive processes results in negative stimulus and response outcome expectancies, creating a vicious circle (2). Certain genetic traits and aspects of personality may reinforce the arousal response further. This situation causes homeostatic instability in itself, establishing another vicious circle (3). In addition, the arousal response may eventually become associated with neutral events, such as moderate physical activity, through the process of classical conditioning (4). We propose that these mechanisms altogether elicit a state of sustained arousal (5).

The mechanism leading to sustained arousal in CFS might be hypothesized from stress theory (Figure 2). Infections, which commonly trigger CFS, generally elicit a normal arousal response [45]. Comparable arousal responses can also be elicited by critical life events and perceived chronic difficulties [20], which have been associated with CFS outbreak (Table 1). Thus, a common characteristic of CFS precipitating factors seem to be their long-lasting character, which – according to CATS – may cause a comparably prolonged arousal response [19].

However, this arousal response might be insufficient in solving the initial problem. An attempt of compensation would be to generate a stronger one. As there is no apparent solution to the individual, such attempts might be perceived as inadequate, resulting in negative stimulus and response outcome expectancy. Thus, a vicious circle is established, as the evaluation of the arousal response depends upon expectancies: negative expectations reinforce the arousal response [19]. This inappropriate learning process can be strengthened by attentiveness, corresponding with reports of increased focus on bodily sensations in CFS [5]. Increased worry about coping abilities is also suggested to be a risk factor [29], complying with personality traits that might be associated with CFS [4]. Finally, genetic factors might have important impact [19], and recent evidence indicates that certain polymorphisms in autonomic and endocrine effector systems are associated with CFS [3].

Therefore, when certain prerequisites are met, the arousal response might be strongly and paradoxically reinforced. This, in turn, may counteract homeostasis rather than restoring it, resulting in another vicious circle. Similar phenomena are demonstrated in hyperventilation, where an unpleasant experience triggers a mutual amplifying cascade of arousal response and unstable respiratory homeostasis, resulting in grossly abnormal blood gas levels [46]. A parallel would be that CFS patients maintain arousal in their pursuits to gain control over their own arousal response.

When the initial triggering factor subsides, classical conditioning may lead to associations between the arousal response and common neutral stimuli [47], like moderate physical activity. Therefore, inappropriate arousal may be precipitated in numerous situations.

We suggest that these mechanisms altogether can elicit a state of sustained arousal.

### Potential consequences of sustained arousal in CFS

**Figure 3:**
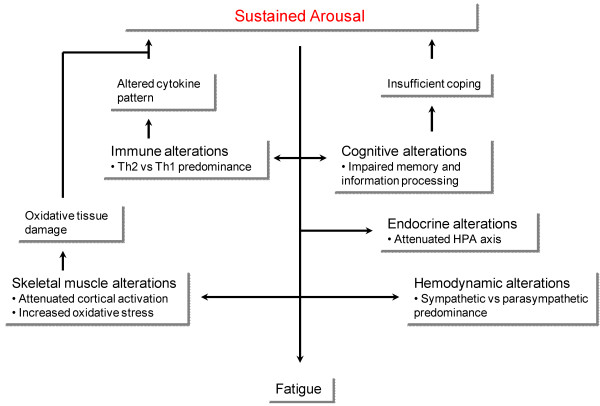
**Proposed model on the consequences of sustained arousal in CFS**. Sustained arousal may cause alterations of immunity, skeletal muscle, cognitions, endocrine function and hemodynamics. Some of these alterations may in turn establish vicious circles due to altered cytokine pattern, oxidative tissue damage and insufficient coping. Finally, sustained arousal might be directly responsible for the experience of fatigue in these patients.

Sustained arousal might have detrimental effects, as evident from stress research experiments (Figure 3). Such consequences correspond well with empirical findings in CFS patients (Table 1). The hemodynamic alterations have been presented above. Below, we shall substantiate our model discussing sustained arousal consequences in other organ systems in relation to CFS research evidence.

A broad range of distressing events, including psychosocial challenges, have an impact on the immune system, such as attenuated cellular immunity and a tendency towards reactivation of latent virus infections [48]. Some of these effects might be attributed to increased sympathetic nerve activity and heightened levels of catecholamines, which in general promote a shift towards Th2 immune responses at the cost of Th1 immune responses [49], complying with findings among CFS patients [2]. Thus, immune dysfunction in CFS may be regarded an epiphenomenon rather than a causal factor [50]. Furthermore, the findings of increased activity of intracellular microorganisms in CFS patients, most convincingly reported for enteroviruses [7], are explained by reactivation of latent infections due to immunological alterations [51]. Although not a primary abnormality according to the sustained arousal model, immune dysfunction in CFS may contribute to vicious circles. For instance, catecholamines stimulate CNS secretion of the Th2-cytokine IL-6 [49], which in turn influences centers involved in arousal responses [52].

An arousal response has profound endocrine consequences, mainly influencing plasma levels of adrenal hormones [20]. Whereas short-lasting arousal activates the HPA axis and increases levels of glucocorticoids, sustained arousal might have the opposite effect [53]. Similar endocrine changes have been documented in CFS [12], and thus seem coherent with our postulate of sustained arousal.

Arousal responses also include behavioral changes, which seem to be mediated – at least in part – by catecholaminergic neurotransmission to brain motor areas [54]. Accordingly, if CFS is resulting from a state of sustained arousal, disturbances of locomotor control will follow [14]. Peripheral interactions between arousal responses and motoric systems might also contribute to skeletal muscle dysfunction in CFS. First, catecholamines strongly influence the excitability of striated muscle cell membrane [55], probably explaining why psychological challenges alters EMG records of skeletal muscle [56]. Similar findings were reported by Jammes and co-workers in CFS patients [16]. Second, catecholamine outflow during an arousal response promote production of free oxygen radicals in striated muscles [57], eventually causing myocyte damage [58]. Corresponding muscular pathology has been reported among CFS patients [16], as well as increased oxidative load in general [15]. Such deleterious effects may in turn enhance resting sympathetic outflow through ordinary reflex mechanisms [59], thus establishing a vicious circle.

Prolonged arousal has a negative influence on memory and information processing [60]. Similar cognitive dysfunctions are found among CFS patients [17,18], giving further support to a hypothesis of sustained arousal. Such dysfunctions may be reinforced through mechanisms of conditioning, establishing negative response outcome expectancies, possibly explaining why patients evaluate their cognitive abilities as even lower than they really are [61]. According to CATS, perceived impairment may be a stronger predictor of outcome than the 'real' impairment, resulting in a self-fulfilling prophecy [19].

Yet the question remains to explain CFS patients' experience of overwhelming fatigue as an effect of sustained arousal. The processes underlying physiologic as well as pathological fatigue are still largely unknown. However, there are several indications of a potential association between fatigue and sustained arousal. Painful and inescapable stimuli increase the serotonin (5-HT) neurotransmission in different brain stem and limbic areas in rats, such as the raphe nucleus [62] and the amygdala [63]. Comparable experiments have demonstrated a concomitant lowering of CRH levels in the hypothalamus [64]. Similar alterations in serotonin and CRH neurotransmission have been reported in CFS patients [65,66], and in other conditions of fatigue [67]. Hence, these two neurotransmitters constitute a possible link between sustained arousal and fatigue.

In addition, more indirect relation involving the immune system might be important. As outlined above, sustained arousal can explain increased production of IL-6 and other Th2 cytokines, which in turn might promote the experience of fatigue through a direct action on the brain stem [67,68]. Finally, CATS suggests a relation between sustained arousal and fatigue at the psychological level, recalling avoidance behavior as a hallmark of arousal responses [19,20]: When a diverse range of stimuli elicit arousal, fatigue and the corresponding functional impairment constitutes an apparently effective way of avoiding them.

## Discussion

### What is known from before – what does the CFS sustained arousal model add?

The model we have presented corresponds with other, recently presented models of CFS disease mechanisms. Altered sympathetic nervous activity at rest and during different challenges has been regarded a key feature by some researchers, but not interpreted within the frame of stress theory [69,70]. Although focusing primarily on a cognitive behavioral model of medically unexplained symptoms in general and CFS in particular, the importance of homeostatic dysregulation and perpetuating vicious circles has been recognized in recent papers [71,72]. Likewise, Gupta outlined the possibility of conditioned responses, but confined this process to the amygdala and emphasized a close relation to the emotional state of fear [73]. In fibromyalgia, a stress model related to ours was promoted by von Houdenhove [74]. Furthermore, the sustained arousal model shares several basic aspects with recently proposed models for the overtraining phenomenon in sports medicine [75], the posttraumatic stress disorder [76], and subjective health complaints [30]

The CFS sustained arousal model holds the capacity to explain *all *main findings concerning CFS disease mechanisms within a unifying theoretical framework. Thus, it represents a synthesis where several important features are included. First, complying with recent cognitive behavioral and self-regulation models [71,72], the sustained arousal model allows heterogeneity of causal factors among individuals. Sustained arousal is seen as a 'common pathway' for the origin of the cluster of CFS symptoms, whereas the combination of predisposing, precipitating and perpetuating factors may vary from patients to patient. Second, the model allows symptom severity to be relative to the extent of distressing challenges; CFS is regarded a kind of maladaptation between stimuli and responses. Third, the model implies a biopsychosocial construct, opposing a fundamental dichotomy between bodily and mental processes, while acknowledging the impact of cognitive processing on physiological responses. Finally, the sustained arousal model seems to possess a kind of 'face validity' when appraised in relation to patients' uniform experience of an inability to respond properly to the physical and mental challenges of daily life [77].

In principle, the arousal response is general and quite non-specific. However, genetic factors, early experiences, personality traits, attributions, and beliefs may all contribute to the specificity of complaints developed by each individual [71]. Likewise, sensitization has been suggested as an underlying mechanism for a number of different conditions; whether these should be lumped together or regarded as distinct entities (such as CFS, irritable bowel syndrome, etc), remains a matter of debate [30].

### Suggestions for further research

Although the assumptions of the sustained arousal model cannot be proved per se, it allows for deduction of testable hypotheses. Below, we suggest a selected sample:

• *Certain polymorphisms of genes whose products are involved in arousal responses are more common among CFS patients than healthy controls*. This could explain the hereditary predisposition for CFS. A search for polymorphisms should be governed by updated knowledge of the genetic basis for normal arousal responses.

• *Infections associated with CFS elicit a more comprehensive and long-lasting arousal response than other infections of similar clinical presentation*. This is to be expected if certain infections are more prone to establish a conditioned arousal response. For instance, patients with verified acute EBV infection might be compared with patients suffering from acute viral pharyngitis of other origin. Autonomic and endocrine reactions to different experimental challenges could be used as markers of an arousal response.

• *When exposed to physical or mental challenges, perfusion, metabolism and transmitter activity in brain areas responsible for arousal responses differs among CFS patients and controls*. Of particular interest are the serotoninergic pathways in the brain stem and limbic structures. Modern neuroimaging technology makes such studies feasible.

• *The characteristics of autonomic reflexes differ among CFS patients and controls during rest and challenges due to set-point alterations*. Exploring further the characteristics of the homeostatic control circuits in CFS patients might add to the evidence supporting a sustained arousal model. A possible analytic tool would be the mathematical technique of transfer function analyses.

• *Cognitive behavioral therapy (CBT) specifically designed to abolish sustained arousal is more effective in the treatment of CFS than unspecific cognitive behavioral therapy*. CBT is of proven value in CFS [1]; to our knowledge, however, the effectiveness of different approaches has not been subjected to research. The design should be randomized and controlled.

• *Pharmaceutical inhibition of brain centers eliciting the arousal response will improve the functional abilities of CFS patients and normalize the hemodynamics in distressing situations*. Clonidine, an agonist to the inhibitory alfa_2_-adrenoceptor, attenuates central sympathetic outflow by an effect on brain stem centers which also constitutes fundamental elements of the arousal response [20]. Thus, this drug – which has well-known antihypertensive and some analgesic properties – might be beneficial in CFS; as yet, no systematic trials have been carried out.

### Concluding remarks

In this article, we have applied CATS and related stress theories to propose the concept of sustained arousal as a coherent and integrative model of disease mechanisms in CFS. This model opposes the reductionist approach where explanations from psychology, neurology immunology and other areas are seen as competing rather than complementary. As stated by Manu [78], page 173: «... more than any other issue in contemporary medicine, chronic fatigue syndrome reflects the unresolved conflict between the mechanistic and the biopsychosocial construct of illness». This warning should guide further research in the field.

## Abbreviations

CFS: Chronic fatigue syndrome; CATS: Cognitive activation theory of stress; HPA-axis: Hypothalamus-pituitary-adrenal-axis; CNS: Central nervous system; CRH: Corticotropin-releasing hormone; CBT: Cognitive behavioral therapy.

## Competing interests

The authors declare that they have no competing interests.

## Authors' contributions

VBW conceived of the theory and drafted the manuscript. HRE and KM elaborated on the theory and helped to draft the manuscript. All authors have read and approved the final manuscript.

## Authors' information

VBW has conducted research projects regarding autonomic control in CFS, and is currently head of the Unit for Chronic fatigue syndrome at Division of Paediatrics, Rikshospitalet University Hospital, Oslo, Norway. HRE has published extensively on the Cognitive activation theory of stress, and is professor at the Dept. of Psychology, University of Bergen, Norway. KM has conducted clinical research on CFS and heads the national research network 'CFS in theory and practice'.

## References

[B1] Prins JB, Meer JW van der, Bleijenberg G (2006). Chronic fatigue syndrome. Lancet.

[B2] Cho HJ, Skowera A, Cleare A, Wessely S (2006). Chronic fatigue syndrome: an update focusing on phenomenology and pathophysiology. Curr Opin Psychiatry.

[B3] Goertzel BN, Pennachin C, Coelho LS, Gurbaxani B, Maloney EM, Jones JF (2006). Combination of single nucleotide polymorphisms in neuroendocrine effector and receptor genes predict chronic fatigue syndrome. Pharmacogenomics.

[B4] Kato K, Sullivan PF, Evengård B, Pedersen NL (2006). Permorbid predictors of chronic fatigue. Arch Gen Psychiatry.

[B5] Heijmans MJ (1998). Coping and adaptive outcome in chronic fatigue syndrome: importance of illness cognitions. J Psychosom Res.

[B6] Hickie I, Davenport T, Wakefield D, Vollmer-Conna U, Cameron B, Vernon SD, Reeves WC, Lloyd A (2006). Post-infective and chronic fatigue syndromes precipitated by viral and non-viral pathogens: prospective cohort study. BMJ.

[B7] Chia JKS (2005). The role of enterovirus in chronic fatigue syndrome. J Clin Pathol.

[B8] Theorell T, Blomkvist V, Lindh G, Evengard B (1999). Critical life events, infections, and symptoms during the year preceding chronic fatigue syndrome (CFS): an examination of CFS patients and subjects with a nonspecific life crisis. Psychosom Med.

[B9] Hatcher S, House A (2003). Life events, difficulties and dilemmas in the onset of chronic fatigue syndrome: a case-control study. Psychol Med.

[B10] Bou-Holaigah I, Rowe PC, Kan JS, Calkins H (1995). The relationship between neurally mediated hypotension and the chronic fatigue syndrome. JAMA.

[B11] Stewart JM (2000). Autonomic nervous system dysfunction in adolescents with postural orthostatic tachycardia syndrome and chronic fatigue syndrome is characterized by attenuated vagal baroreflex and potentiated sympathetic vasomotion. Pediatr Res.

[B12] Cleare AJ (2003). The neuroendocrinology of chronic fatigue syndrome. Endocr Rev.

[B13] Fulcher KY, White PD (2000). Strength and physiological response to exercise in patients with chronic fatigue syndrome. J Neurol Neurosurg Psychiatry.

[B14] Schillings ML, Kalkman JS, Werf SP van der, van Engelen BG, Bleijenberg G, Zwarts MJ (2004). Diminished central activation during maximal voluntary contraction in chronic fatigue syndrome. Clin Neurophysiol.

[B15] Kennedy G, Spence VA, McLaren M, Hill A, Underwood C, Belch JJF (2005). Oxidative stress levels are raised in chronic fatigue syndrome and are associated with clinical symptoms. Free Radical Biol Med.

[B16] Jammes Y, Steinberg JG, Mambrini O, Bregeon F, Delliaux S (2005). Chronic fatigue syndrome: assessment of increased oxidative stress and altered muscle excitability in response to incremental exercise. J Intern Med.

[B17] De Luca J, Johnson SK, Ellis SP, Natelson BH (1997). Cognitive functioning is impaired in patients with chronic fatigue syndrome devoid of psychiatric disease. J Neurol Neurosurg Psychiatry.

[B18] Michiels V, Cluydts R (2001). Neuropsychological functioning in chronic fatigue syndrome: a review. Acta Psychiatr Scand.

[B19] Ursin H, Eriksen HR (2004). The cognitive activation theory of stress. Psychoneuroendocrinology.

[B20] Goldstein DS (2001). The autonomic nervous system in health and disease.

[B21] McEwen BS (1998). Protective and damaging effects of stress mediators. N Engl J Med.

[B22] Levine S, Ursin H, Brown MR, Rivier C, Koob G (1991). What is stress?. Stress Neurobiology and Neuroendocrinology.

[B23] Sokolov YN (1963). Perception and the conditioned reflex.

[B24] Dickinson A, Klein SB, Mowrer RR (1989). Expectancy theory in animal conditioning. Contemporary learning theories: Pavlovian conditioning and the status of learning theory.

[B25] Steriade M (1996). Arousal: Revisiting the reticular activating system. Science.

[B26] Bolles RC (1972). Reinforcement, expectancy and learning. Psychol Rev.

[B27] Thompson RF, Spencer WA (1966). Habituation: A model phenomenon for the study of neuronal substrates of behavior. Psychol Rev.

[B28] Kandel ER, Schwartz JH, Jessell TM (2000). Principles of Neural Science.

[B29] Brosschot JF, Gerin W, Thayer JF (2006). The perseverative cognition hypothesis: a review of worry, prolonged stress-related physiological activation, and health. Psychosom Res.

[B30] Eriksen HR, Ursin H (2004). Subjective health complaints, sensitization and sustained cognitive activation (stress). J Psychosom Res.

[B31] Staud R, Smitherman ML (2002). Peripheral and central sensitization in fibromyalgia: pathogenic role. Curr Pain Headache Rep.

[B32] Wilhelmsen I (2002). Somatization and functional dyspepsia. Scan J Psychol.

[B33] Wyller VB, Due R, Saul JP, Amlie JP, Thaulow E (2007). Usefulness of an abnormal cardiovascular response during low-grade head-up tilt-test for discriminating adolescents with chronic fatigue from healthy controls. Am J Cardiol.

[B34] Wyller VB, Saul JP, Walløe L, Thaulow E (2008). Sympathetic cardiovascular control during orthostatic stress and isometric exercise in adolescents with chronic fatigue syndrome. Eur J Appl Physiol.

[B35] Wyller VB, Godang K, Mørkrid L, Saul JP, Thaulow E, Walløe L (2007). Abnormal thermoregulatory responses in adolescents with chronic fatigue syndrome: relation to clinical symptoms. Pediatrics.

[B36] Wyller VB, Barbieri R, Thaulow E, Saul JP (2008). Enhanced vagal withdrawal during mild orthostatic stress in adolescents with chronic fatigue. Ann Noninvasive Electrocardiol.

[B37] Wyller VB, Saul JP, Amlie JP, Thaulow E (2007). Sympathetic predominance of cardiovascular regulation during mild orthostatic stress in adolescents with chronic fatigue. Clin Physiol Funct Imaging.

[B38] Kimmerly DS, Shoemaker JK (2002). Hypovolemia and neurovascular control during orthostatic stress. Am J Physiol Heart Circ Physiol.

[B39] Convertino VA, Low PA (1997). Conditions of reduced gravity. Clinical autonomic disorders.

[B40] Tirelli U, Chierichetti F, Tavio M, Simonelli C, Bianchin G, Zanco P, Ferlin G (1998). Brain positron emission tomography (PET) in chronic fatigue syndrome: preliminary data. Am J Med.

[B41] Wyller VB (2007). The pathophysiology of chronic fatigue syndrome in adolescents.

[B42] Timmers HJ, Wieling W, Soetekouw PM, Bleijenberg G, Meer JW van der, Lenders JW (2002). Hemodynamic and neurohumoral responses to head-up tilt in patients with chronic fatigue syndrome. Clin Auton Res.

[B43] Lucini D, Di Fede G, Parati G, Pagani M (2005). Impact of chronic psychosocial stress on autonomic cardiovascular regulation in otherwise healthy subjects. Hypertension.

[B44] Di Micco JA, Sarkar S, Zaratskaia MV, Zaretsky DV (2006). Stress-induced cardiac stimulation and fever: common hypothalamic origins and brainstem mechanisms. Auton Neurosci.

[B45] Shanks N, Harbuz MS, Jessop DS, Perks P, Moore PM, Lightman SL (1998). Inflammatory disease as chronic stress. Ann N Y Acad Sci.

[B46] Gorman JM, Uy J (1987). Respiratory physiology and pathological anxiety. Gen Hosp Psychiatry.

[B47] Overmier JB (2002). Sensitization, conditioning, and learning: can they help us understand somatization and disability?. Scand J Psychol.

[B48] Glaser R, Kiecolt-Glaser JK (1998). Stress-associated immune modulation: relevance to viral infections and chronic fatigue syndrome. Am J Med.

[B49] Elenkov IJ, Wilswe RL, Chrousos GP, Vizi ES (2000). The sympathetic nerve – an integrative interface between two supersystems: the brain and the immune system. Pharmacol Rev.

[B50] Clauw DJ, Chrousos GP (1997). Chronic pain and fatigue syndromes: overlapping clinical and neuroendcrine features and potential pathogenic mechanisms. Neuroimmunomodulation.

[B51] Komaroff AL, Gupta S, Salit IE, Ablashi DV, Faggioni A, Keiger GRF (1989). Post-viral fatigue syndrome. Epstein-Barr virus and human disease.

[B52] Lenczowski MJ, Bluthe RM, Roth J, Rees GS, Rushforth DA, van Dam AM, Tilders FJ, Dantzer R, Rothwell NJ, Luheshi GN (1999). Central administration of rat IL-6 induces HPA activation and fever but not sickness behaviour in rats. Am J Physiol.

[B53] Yehuda R, Giller EL, Southwick SM, Lowy MT, Mason JW (1991). Hypothalamic-pitutitary-adrenal dysfunction in posttraumatic stress disorder. Biol Psychiatry.

[B54] Stone EA, Yan L, Ahsan R, Lehmann ML, Yeretsian J, Quartermain D (2006). Role of CNS alpha1-adrenoceptor activity in central fos responses to novelty. Synapse.

[B55] Hansen AK, Clausen T, Nielsen OB (2005). Effects of lactic acid and catecholamines on contractility in fast-twitch muscles exposed to hyperkalemia. Am J Physiol Cell Physiol.

[B56] Rissen D, Melin B, Sandsjø L, Dohns I, Lundberg U (2000). Surface EMG and psychophysiological stress reactions in women during repetitive work. Eur J Appl Physiol.

[B57] Dhalla NS, Temsah RM, Netticadan T (2000). Role of oxidative stress in cardiovascular diseases. J Hypertens.

[B58] Burniston JG, Tan LB, Goldspink DF (2005). Beta2-adrenergic receptor stimulation in vivo induces apoptosis in the rat heart and soleus muscle. J Appl Physiol.

[B59] Ray CA, Mark AL (1993). Augmentation of muscle sympathetic nerve activity during fatiguing isometric leg exercise. J Appl Physiol.

[B60] Luoto S, Taimela S, Hurri H, Alaranta H (1999). Mechanisms explaining the association between low back trouble and deficits in information processing. A controlled study with follow-up. Spine.

[B61] Metzeger FA, Denney DR (2002). Perception of cognitive performance in patients with chronic fatigue syndrome. Ann Behav Med.

[B62] Maswood S, Barter JE, Watkins LR, Maier SF (1998). Exposure to inescapable but not escapable shock increases extracellular levels of 5-HT in the dorsal raphe nucleus of the rat. Brain Res.

[B63] Amat J, Matus-Amat P, Watkins LR, Maier SF (1998). Escapable and inescapable stress differentially alter extracellular levels of 5-HT in the basolateral amygdala of the rat. Brain Res.

[B64] Davis JM, Bailey SP (1997). Possible mechanisms of central nervous fatigue during exercise. Med Sci Sport Exerc.

[B65] Cleare AJ, Bearn J, Allain T, McGregor A, Wessely S, Murray RM, O'Keane V (1995). Contrasting neuroendocrine responses in depression and chronic fatigue syndrome. J Affect Disord.

[B66] Tanriverdi F, Karaca Z, Unluhizarci K, Kelestimur F (2007). The hypothalamo-pituitary-adrenalin axis in chronic fatigue syndrome and fibromyalgia syndrome. Stress.

[B67] Swain MG (2000). Fatigue in chronic disease. Clin Sci.

[B68] Dantzer R (2005). Somatization: a psychoneuroimmune perspective. Psychoneuroendocrinology.

[B69] Naschitz J (2004). Dysautonomia in chronic fatigue syndrome: facts, hypotheses, implications. Med Hypotheses.

[B70] Pagani M, Lucini D (1999). Chronic fatigue syndrome: a hypothesis focusing on the autonomic nervous system. Clin Sci.

[B71] Deary V (2008). A precarious balance: Using self-regulation model to conceptualize and treat chronic fatigue syndrome. Br J Health Psychol.

[B72] Deary V, Chalder T, Sharpe M (2007). The cognitive behavioural model of medically unexplained symptoms: A theoretical and empirical view. Clin Psychol Rev.

[B73] Gupta A (2002). Unconscious amygdalar fear conditioning in a subset of chronic fatigue syndrome patients. Med Hypotheses.

[B74] Von Houdenhove B, Egle UT (2004). Fibromyalgia: a stress disorder? Piecing the biopsychosocial puzzle together. Psychother Psychosom.

[B75] Angeli A, Minetto M, Dovio A, Paccotti P (2004). The overtraining syndrome in athletes: a stress-related disorder. J Endocrinol Invest.

[B76] Bedi US, Arora R (2007). Cardiovascular manifestations of posttraumatic stress disorder. J Natl Med Assoc.

[B77] Soderlund A, Skoge AM, Malterud K (2000). I could not lift my arm holding the fork...". Living with chronic fatigue syndrome. Scand J Prim Health Care.

[B78] Manu P (2000). Chronic fatigue syndrome: the fundamentals still apply. Am J Med.

